# Assessing the relationship between malaria incidence levels and meteorological factors using cluster-integrated regression

**DOI:** 10.1186/s12879-024-09570-z

**Published:** 2024-07-03

**Authors:** Miracle Amadi, K. K. W. Hasitha Erandi

**Affiliations:** 1 LUT School of Engineering Science, Lappeenranta-Lahti University of Technology LUT, Lappeenranta, FI-53850 Finland; 2 Department of Mathematics, University of Colombo, Colombo, 00300 Sri Lanka

**Keywords:** Malaria modeling, Regression, Climate, Incidence clustering, Marginal effects

## Abstract

This paper introduces a novel approach to modeling malaria incidence in Nigeria by integrating clustering strategies with regression modeling and leveraging meteorological data. By decomposing the datasets into multiple subsets using clustering techniques, we increase the number of explanatory variables and elucidate the role of weather in predicting different ranges of incidence data. Our clustering-integrated regression models, accompanied by optimal barriers, provide insights into the complex relationship between malaria incidence and well-established influencing weather factors such as rainfall and temperature.

We explore two models. The first model incorporates lagged incidence and individual-specific effects. The second model focuses solely on weather components. Selection of a model depends on decision-makers priorities. The model one is recommended for higher predictive accuracy. Moreover, our findings reveal significant variability in malaria incidence, specific to certain geographic clusters and beyond what can be explained by observed weather variables alone.

Notably, rainfall and temperature exhibit varying marginal effects across incidence clusters, indicating their differential impact on malaria transmission. High rainfall correlates with lower incidence, possibly due to its role in flushing mosquito breeding sites. On the other hand, temperature could not predict high-incidence cases, suggesting that other factors other than temperature contribute to high cases.

Our study addresses the demand for comprehensive modeling of malaria incidence, particularly in regions like Nigeria where the disease remains prevalent. By integrating clustering techniques with regression analysis, we offer a nuanced understanding of how predetermined weather factors influence malaria transmission. This approach aids public health authorities in implementing targeted interventions. Our research underscores the importance of considering local contextual factors in malaria control efforts and highlights the potential of weather-based forecasting for proactive disease management.

## Introduction

Malaria persists as a significant health concern globally, particularly in sub-Saharan Africa, posing a substantial threat to nearly half of the world’s inhabitants [1–3]. Annually, it contributes to a minimum of one million fatalities, with more than $90\%$ of these occurrences transpiring in Africa [4, 5].

Malaria has a significant socioeconomic impact, making it not only a public health concern but also a major contributor to poverty and underdevelopment [6, 7]. Malaria vaccinations are likely insufficient, and despite more than 60 years of research, an effective vaccine that outperforms naturally acquired immunity has yet to be developed [8–11]. This underscores the importance of malaria research for socioeconomic advancement in Africa and globally. In Nigeria, where malaria transmission is widespread, 97% of the population is at risk. In 2019, Nigeria was one of six countries that accounted for 55% of global malaria cases [12]. Factors such as poor sanitation, overcrowded living conditions, and high population density contribute to the prevalence of malaria in Nigeria [13]. Malaria is actively transmitted across all 36 states of Nigeria [14]. Despite efforts to increase coverage, the proportion of Insecticide-Treated Net (ITN) usage remains low in many endemic regions [15–17], and the non-significant negative relationship between malaria transmission and ITN coverage is concerning. This appears to be due to the barriers to ITN or Long-Lasting Insecticidal Net (LLIN) utilization which include heat, adverse reactions to the chemicals, unpleasant odors, and cost [18, 19].

Moreover, meteorological factors play a crucial role in driving malaria transmission, influencing the life cycles of both vectors and parasites [20]. Mosquito breeding, which relies on stagnant water accumulation, is directly affected by precipitation levels. While adequate rainfall can create breeding sites, intense rainfall can also impact mosquito habitats [21–23]. Additionally, ambient temperature is another key factor, as temperatures above 20^∘^C are essential for the development of the Plasmodium parasite. This is particularly true for Plasmodium falciparum, the most common malaria parasite in tropical regions [24].

Numerous studies have explored the intricate connection between malaria incidence and various meteorological factors [25–29]. For instance, Gunda et al. [28] conducted an assessment of malaria incidence and its correlation with weather variables in three rural districts in Sub-Saharan Africa (SSA), highlighting a significant association with precipitation and mean temperature at specific lag periods. Similarly, Akinbobola and Omotosho [29] examined the relationship between weather variables and reported malaria cases in two stations from different geopolitical zones in Nigeria. Their study revealed a notable increase in malaria cases associated with changes in weather variables. Specifically, they found that rainfall and humidity had positive associations with the incidence of malaria, while maximum temperature exhibited both inverse and direct relationships, depending on the region under consideration. Further, the study in [30] found that malaria incidence in Nigeria is significantly influenced by environmental factors such as rainfall, temperature, and proximity to water. The research found that higher rates of malaria are associated with increased rainfall and temperatures. The spatio-temporal study identified specific hotspots, facilitating the development of targeted intervention strategies. Additionally, the study indicates that malaria is more prevalent in the northern regions and rural areas compared to the southern and urban regions. Moreover, recent research in Uganda has demonstrated strong associations between malaria incidence and climate variables, indicating a positive correlation with rainfall as well as average temperature [29]. Similarly, a decade-long investigation of regional and temporal patterns of malaria incidence in Mozambique found a higher risk when maximum temperatures exceeded $28^{\circ }$C and humidity reached 95% [31]. In addition, regions such as Ethiopia and Senegal have shown similar spatial relationships between climatic variability, such as rainfall, and malaria occurrence [32]. These findings underscore the significant influence of climatic factors on malaria prevalence within endemic environments.

**Fig. 1:**
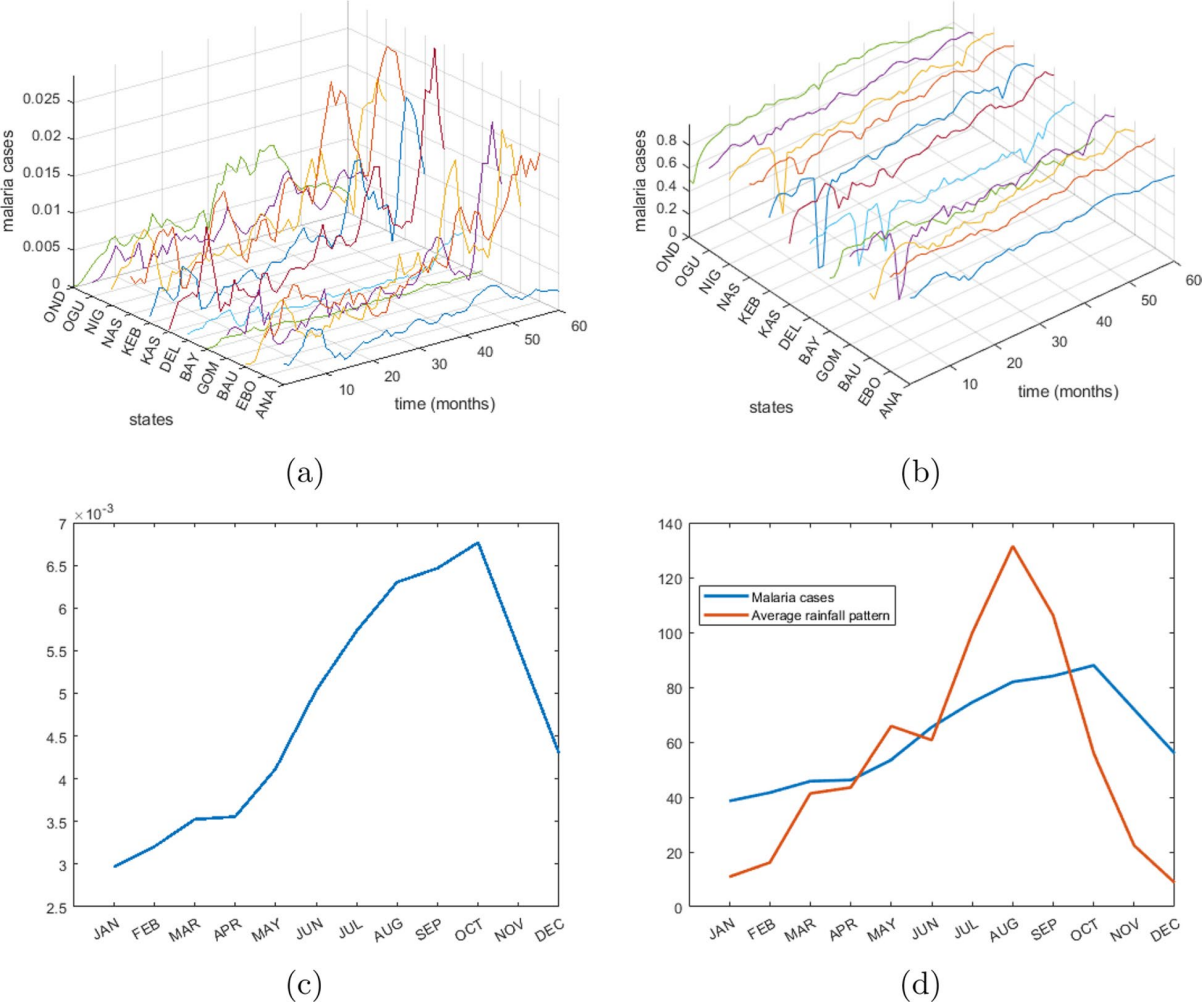
**a** Monthly malaria density data. **b** Normalized malaria data. **c** Average rainfall data. **d** Malaria cases versus average rainfall pattern

Driven by the evident seasonal fluctuations in malaria prevalence, particularly with a notable percentage of cases occurring during wet seasons [33–35] (see also Fig. 1b), this study investigates the relationship between panel malaria incidence data from Nigeria and meteorological variables. Our main objective is to discover the incidence ranges that are most efficiently predicted by meteorological factors, contrasting the traditional approach of predicting weather components based on incidence levels. To achieve this, we develop a clustering-integrated multiple regression model for monthly panel data, incorporating meteorological factors such as rainfall and average temperature. By categorizing incidence data into distinct clusters using a clustering method [36–39], we improve model fitting and identify clusters where meteorological factors inadequately predict incidence. By varying clustering barriers to minimize mean squared error, we aim to optimize model performance. This approach addresses challenges posed by limited data availability, (particularly, the additional confounding factors) in developing countries, by proposing a clustering strategy to enhance modeling accuracy. Additionally, utilizing panel data from different states across Nigeria enriches our analysis.

### Data and study area

The dataset used in this study comprises monthly reports of malaria cases over five years (2014-2018), obtained from the National Malaria Elimination Programme, Federal Ministry of Health, Nigeria. The data encompasses all six geo-political zones in Nigeria, with two states representing each zone. This includes a total of twelve states, which collectively represent one-third of Nigeria’s regions: Anambra, Ebonyi, Bauchi, Gombe, Bayelsa, Delta, Kastina, Kebbi, Nassarawa, Niger, Ogun, and Ondo. The visualization of reported malaria cases is depicted in Fig. 1.

To address the disparity in data magnitude between meteorological and incidence data, which may stem from population variations among regions, we employ normalized density data to ensure interpretability and numerical stability. Population data required for normalization are sourced from the demographic statistics bulletin of Nigeria.

**Fig. 2:**
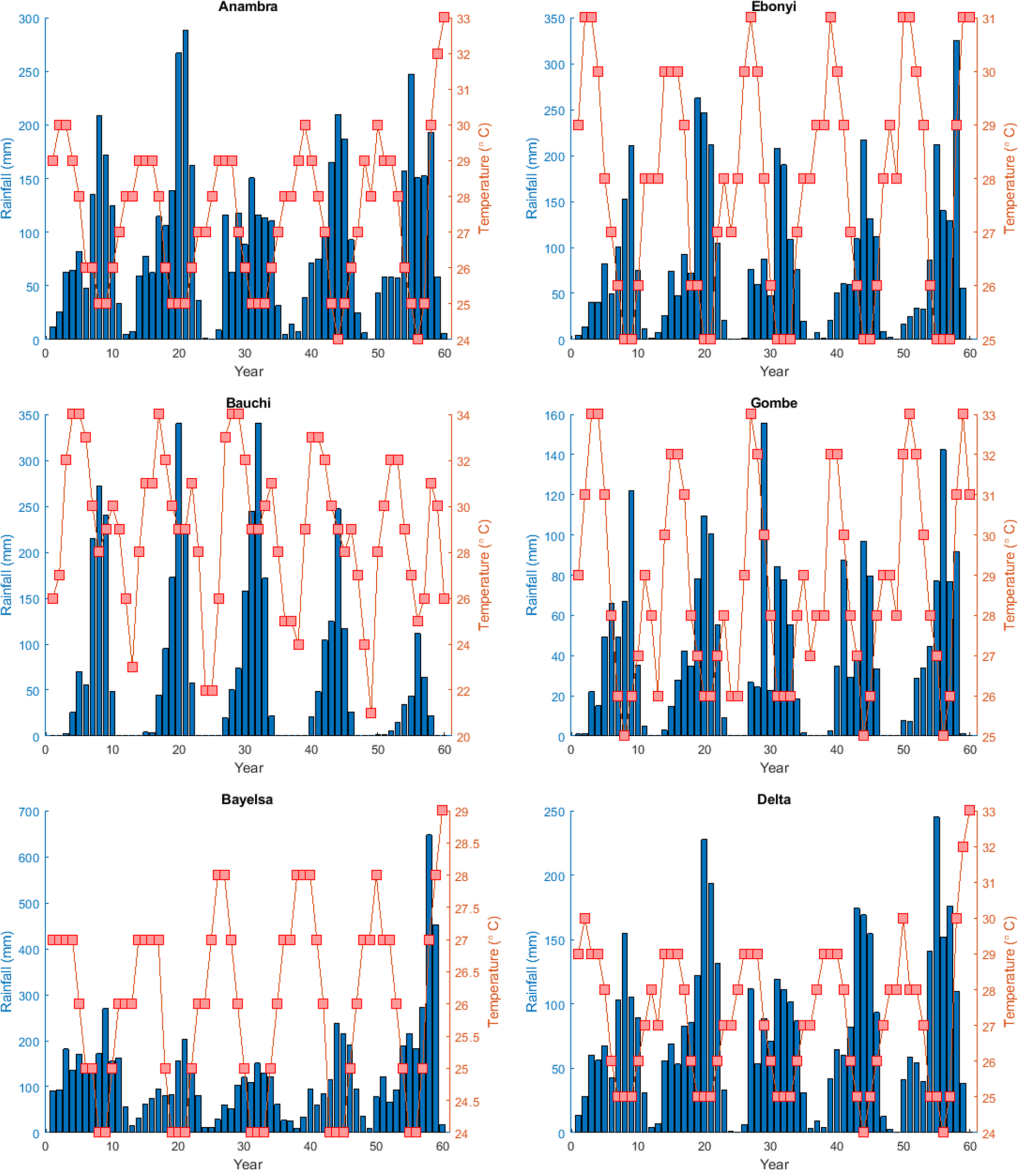
Rainfall and temperature data for different states

**Fig. 3:**
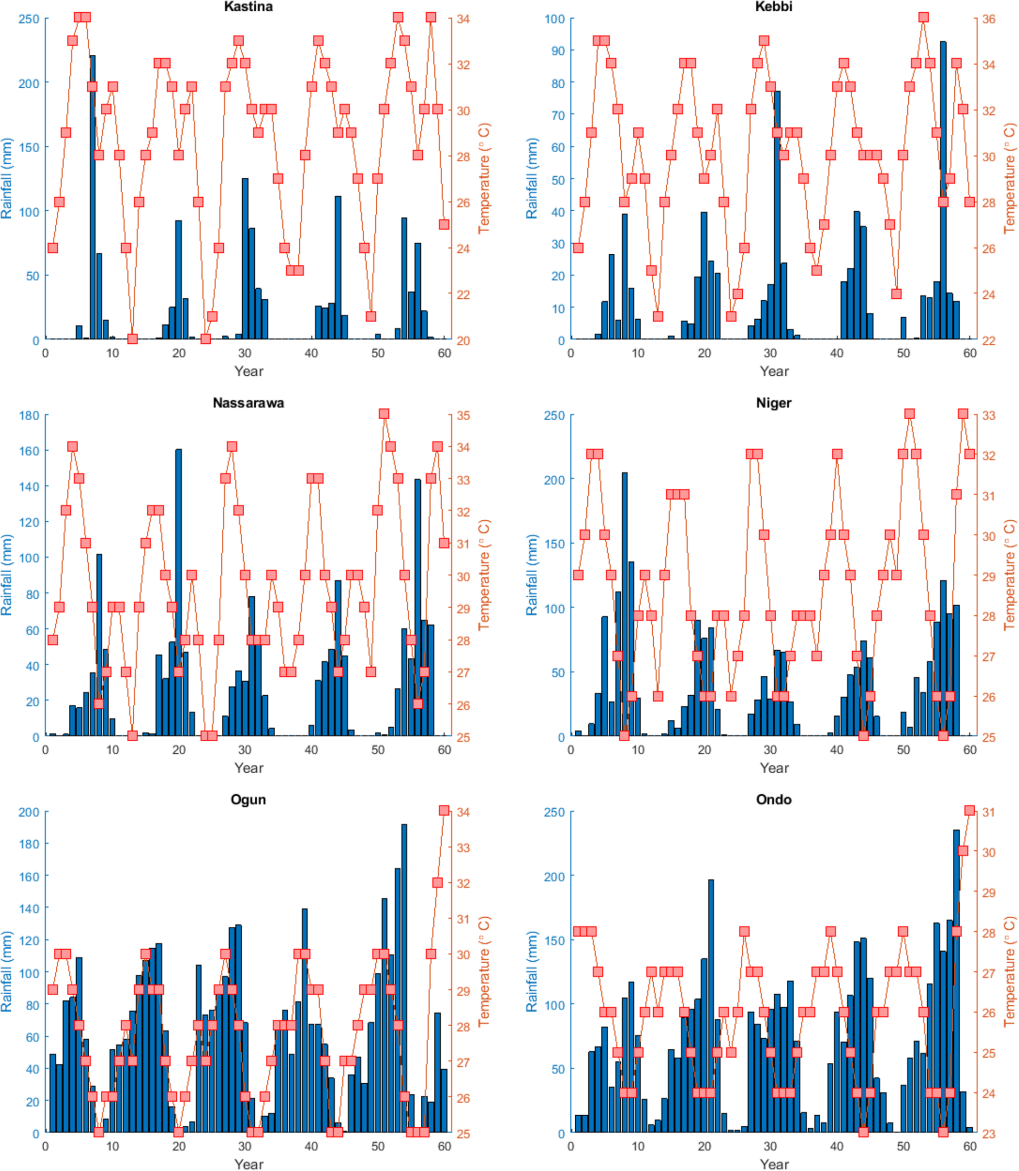
Rainfall and temperature data for different states

The corresponding mean monthly rainfall and temperature data were obtained for each of the aforementioned states from the World Weather Online. These states altogether comprise areas of low and intense temperatures and rainfall. The plot of the climate data is given in Figs. 2 and 3.

In Nigeria, the climate exhibits significant diversity, ranging from tropical conditions in the south to semi-arid conditions in the far north. Precipitation patterns vary accordingly, with the annual rainfall below 500*mm* (20 inches) in the extreme northeast, increasing to 1,000 to 1, 500*mm* (40 to 60 in) in the central region, and exceeding 2, 000*mm* (80 in) in the south, particularly in the far southeast. Temperatures also display notable fluctuations across different climatic zones.

In the northern regions, winters are warm and dry, with daytime temperatures soaring to uncomfortable levels of up to 40^∘^C (104^∘^F), while nights are generally cool. In hilly areas of the north, temperatures can drop to freezing (0^∘^C or 32^∘^F). From February onwards, temperatures rise across inland areas, reaching scorching levels from March to May, with temperatures often surpassing 40^∘^C (104^∘^F) in the center-north.

Conversely, in the southern regions, temperature increases are more moderate due to the proximity to the ocean and the onset of rain showers earlier in the year. Rainfall intensifies and becomes more frequent, gradually spreading northwards until it affects the entire country by June. This information is sourced from the Nigerian Meteorological Agency (https://nimet.gov.ng/).

Additionally, analysis of average monthly rainfall trends in Fig. 1c, illustrates that the rainy season typically commences between March and April, although variations occur from year to year and from state to state. The peak of rainfall typically occurs in August, followed by a tapering off of the rainy season from September to October, with the dry season prevailing from November onwards. Notably, Fig. 1d highlights a two-month lag between the peak of rainfall and the peak in malaria cases.

## Methods

### Cross-correlations and autocorrelations

We determine the time delays between malaria incidence and meteorological factors prior to their integration into the modeling process. Spearman’s correlation coefficient is employed for this purpose due to its resilience to monotonic transformations across datasets. Using a dataset comprising 60 data points, we fix the last 12 malaria incidence data points and shift 48-month windows of meteorological parameters backward in time for all states. This approach allows us to explore correlations over a full one-year period shift. For each fixed window, correlation coefficients are computed, and the maximum correlation is identified. The corresponding time lags associated with these maximal correlation coefficients are summarized in Table 1.

**Table 1:** Maximum correlation coefficients and associated time lags following Spearman’s cross-correlation analysis between malaria incidence and meteorological variables in 12 selected states in Nigeria

States	Rainfall	Temperature
Anambra	1 mths (0.3419)	6 mths (0.5152)
Ebonyi	3 mths (0.4101)	4 mths (0.3568)
Bauchi	2 mths (0.4455)	5 mths (0.4234)
Gombe	2 mths (0.5241)	6 mths (0.5506)
Bayelsa	1 mths (0.2101)	mths (0.3163)
Delta	1 mths (0.3847)	7 mths (0.2367)
Kastina	2 mths (0.5144)	2 mths (-0.3568)
Kebbi	2 mths (0.4492)	0 mths (-0.2998)
Nassarawa	1 mths (0.5331)	2 mths (-0.4998)
Niger	2 mths (0.4811)	5 mths (0.3314)
Ogun	2 mths (0.5359)	6 mths (0.3569)
Ondo	3 mths (0.4101)	4 mths (0.2568)

Furthermore, auto-correlation analysis of malaria cases is conducted by consolidating spatiotemporal data into a single time series, considering the relatively minor variations in normalized data. Lags up to 6 months from the present are selected, resulting in each covariate augmenting the dataset by 12 times 60 observations. Subsequently, Spearman-rank correlation coefficients are computed, as illustrated in Table 2.

### Panel regression

As mentioned in the Introduction, this study focuses on modeling malaria incidence in Nigeria using rainfall and temperature data obtained from various states across different periods. The data used in this study is categorized as panel data, encompassing both cross-sectional (across states) and time-series dimensions, as it provides insights into individual behavior over time.

Commonly two primary models are employed for panel data analysis; the fixed effects model and the random effects model. Consider the multiple linear regression model for individual $i = 1, \ldots ,N$, observed at various time points $t = 1, \ldots , T$:

1 $$\begin{aligned} y_{it} = \alpha + x^{\prime }_{it} \beta + z^{\prime }_i \gamma + c_i + u_{it} \end{aligned}$$

Here, $y_{it}$ represents the dependent variable, $x^{\prime }_{it}$ denotes a *K*-dimensional row vector of time-varying explanatory variables, $z^{\prime }_i$ signifies a *M*-dimensional row vector of time-invariant explanatory variables (excluding the constant term), $\alpha$ stands for the intercept, $\beta$ represents a *K*-dimensional column vector of parameters, $\gamma$ denotes a *M*-dimensional column vector of parameters, $c_i$ denotes an individual-specific effect, and $u_{it}$ signifies the idiosyncratic error term.

The fixed effects model accommodates for individual-specific effects ($\alpha _i$) that may be correlated with the regressors *x*. In contrast, the random effects model assumes that these individual-specific effects ($\alpha _i$) are distributed independently from the regressors. The selection between the fixed and random effects models is determined using the Hausman test [40, 41]. This test evaluates whether there is a significant difference exists between the fixed and random effects estimators. Specifically, the test statistic is computed solely for the time-varying regressors. If the Hausman test yields an insignificant result, the random effects model is employed. Otherwise, the fixed effects model is preferred [42].

Given the limited availability of incidence data for modeling, utilizing panel data offers specific advantages in uncovering the correlations between malaria incidence and meteorological factors. This stems from several established benefits of employing panel data compared to relying solely on time series or cross-sectional data [40, 43].

One advantage lies in incorporating individual-specific components within the model, which enables addressing heterogeneity across individuals. Integrating this component elucidates correlations among observations over time that are not solely attributable to dynamic trends, thereby mitigating unexplained variability. Additionally, incorporating individual-specific effects helps mitigate the issue of omitted variable bias.

Another advantage, particularly in the time series analysis, is leveraging the available data across individuals to compensate for shorter series lengths, obviating the need for extensive longitudinal data. Consequently, constructing an accurate model becomes feasible by identifying commonalities among individuals.

Conversely, compared to cross-sectional data, panel data’s temporal dimension enhances estimation precision through additional temporal data points.

### Incidence-weather relation:(without clustering strategy)

Let *i* and *j* represent the state and time indices respectively, where $i\in \{1,\cdots ,S=12\}$ and $j\in \{1,\cdots ,N\}$. Our strategy for modeling the monthly malaria cases in the 12 chosen Nigerian states involves directly linking collected variables. These variables consist of current (lag-0) reported cases $C =(c_{ij})$, cases reported in the preceding six months (lag-1, $\cdots$, lag-6) from the current period $C_{-1} = (c_{i,j-1}),\cdots ,C_{-6} =(c_{i,j-6})$, lagged monthly rainfall $R =\mathbbm {1}_{S}\otimes (r_{j-lag(i)})$, and lagged monthly temperature $T =\mathbbm {1}_{S}\otimes (t_{j-lag(i)})$; where *lag*(*i*) corresponds to the cross-correlation outcome in Table 1. The symbols $\mathbbm {1}_{S}$ and $\otimes$ denote the column vector of size *S* with entries being 1, and the Kronecker product between two matrices respectively. The total number of observations is the length of the entire time window minus the maximum autoregressive lag. Let $\beta _0$ denote the intercept and $\beta _{\text {ind}}=(\beta _1,\cdots ,\beta _{S-1})$ represent the individual-specific effects (reduced by one term to prevent linear dependence with the intercept). Further, $\beta _{-i}$ (for $i=1,\cdots ,6$) signify the marginal effects of the lagged incidence cases while $\beta _R$, $\beta _T$ and $\varepsilon =(\varepsilon _{ij})$ represent the marginal effect of rainfall, marginal effect of temperature and the idiosyncratic error respectively. The direct relationship among these covariates is represented by:

2 $$\begin{aligned} C = \beta _0\mathbbm {1}_{S\times N} + \mathbbm {1}_{N}^{\top }\otimes [\beta _{\text {ind}} \,0]^{\top } + \beta _RR+\beta _TT+\varepsilon . \end{aligned}$$

### Incidence-weather relation:(with clustering strategy)

Clustering is applied to the response data, while the associated explanatory variables are categorized based on the levels of the response data. This technique allows for the selective use of certain explanatory variables to predict a specific response variable, particularly when the number of explanatory variables is limited. The primary objective of clustering is to accurately allocate explanatory variables in scenarios where they may not predict a particular response variable effectively. Therefore, unlike conventional regression approaches, clustering-integrated regression aims to identify the ranges of the response variable that are well predicted by the available explanatory variables. By incorporating additional explanatory variables, this approach can enhance model fitting.

The clustering concept involves categorizing the incidence data into *M* clusters $(\Omega _{k})_{k=1}^M$ separated by barriers $\theta :=(\theta _{k})_{k=1}^{M-1}$. In closed forms, the clusters are defined as $\Omega _k=\{c:\max \{0,\theta _{k-1}\}\le c<\min \{\theta _k,\max _{i,j}c_{ij}\}\}$. Let $\delta _k(C;\theta ):=(\mathbbm {1}_{\Omega _k}c_{ij})$, where $\mathbbm {1}_{\Omega _k}$ denotes the characteristic function and assigning a value of 1 to $c_{ij}$ belonging to $\Omega _k$ or 0 otherwise. Define $R^k=R^k(\theta ):=\delta _k(C;\theta )\circ R$ and $T^k=T^k(\theta ):=\delta _k(C;\theta )\circ T$, where the Hadamard product $\circ$ represents the element-wise multiplication between matrices. These matrices return the original entries of *R* and *T* if their corresponding incidence cases belong to the respective cluster or 0 otherwise. This decomposition ensures that $\sum _kR^k=R$ and $\sum _kT^k=T$. Incorporating clustering, the model (2) is modified as follows:

3 $$\begin{aligned} C = \beta _0\mathbbm {1}_{S\times N} + \mathbbm {1}_{N}^{\top }\otimes \beta _{\text {ind}}^{\top } + \sum _{i=1}^3 \beta ^i_RR^{(i)}+\sum _{i=1}^3 \beta ^i_TT^{(i)}+\varepsilon . \end{aligned}$$

In theory, the number of specified clusters is not limited to a small number, as better fitting can be achieved with more explanatory factors. However, concerns about complexity and interpretability may arise when adopting a large number of clusters. For example, if $R^{(2)}$ is deemed insignificant, it implies that rainfall fails to predict response cases within the range specified by the middle cluster $\Omega _2$. This approach allows such cases to remain “unexplained by rainfall”.

The pooled estimator $\hat{\beta }$ changes with the lower and upper barriers $\theta =(\theta _{\text {l}},\theta _{\text {u}})$, as do $R^k$ and $T^k$. Our objective is to determine the optimal barriers such that the squared error between the data $C=(c_{ij})$ and the model approximation $C[\hat{\beta }](\theta )$ is minimized. Mathematically, this translates to the optimization problem:

4a $$\begin{aligned}{} & {} \min _{\theta }\qquad \qquad \qquad \quad \sum _{i,j}\,(c_{ij}[\hat{\beta }](\theta )-c_{ij})^2\end{aligned}$$

4b $$\begin{aligned}{} & {} \text {subject to}\qquad \qquad \min _{i,j}c_{ij}\le \theta _{\text {l}}\le \theta _{\text {u}}\le \max _{i,j}c_{ij}. \end{aligned}$$

This problem can be solved using optimization techniques such as brute-force or particle swarm optimization methods [44].

### Data management and statistical analysis

The malaria incidence data, being population-driven, underwent normalization to account for population variations across states. Specifically, normalization was performed to standardize the number of cases per 100,000 inhabitants based on the 2006 population census. This intentional normalization aimed to ensure comparability of malaria incidence rates across states, facilitating appropriate comparison with weather components unaffected by population dynamics. Due to the skewed nature of the datasets, and given that the applicability of linear regression necessitates that all datasets are identically and independently distributed (i.i.d.). To enhance compatibility with dummy or coded variables, we normalize the incidence and weather datasets. The incidence data transformation was done using the Johnson SU technique [45]. The transformation is defined as follows:

5 $$\begin{aligned} T(\textbf{x}) = \text {shift} + \text {grad} \cdot \sinh ^{-1}\left( \frac{\textbf{x}}{\text {div}}\right) \end{aligned}$$

where:

- $\text {shift}$ is the shift parameter,
- $\text {grad}$ is the gradient parameter,
- $\text {div}$ is the divisor parameter,
- $\textbf{x}$ represents the data points.

For incidence data transformation, we use the parameters [shift, grad, div]= [0, 0.1, 0.000003]. We employed the logarithmic transformation for rainfall and temperature data and defined as follows:

6 $$\begin{aligned} \text {T}(\textbf{x}) = \log _{10}\left( \text {shift} + \frac{\textbf{x}}{\text {div}}\right) \end{aligned}$$

where:

- $\textbf{x}$ represents the data points.
- $\text {shift}$ is a shift parameter added to the data to ensure all values are positive before applying the logarithm.
- $\text {div}$ is a divisor that scales the data.

**Fig. 4:**
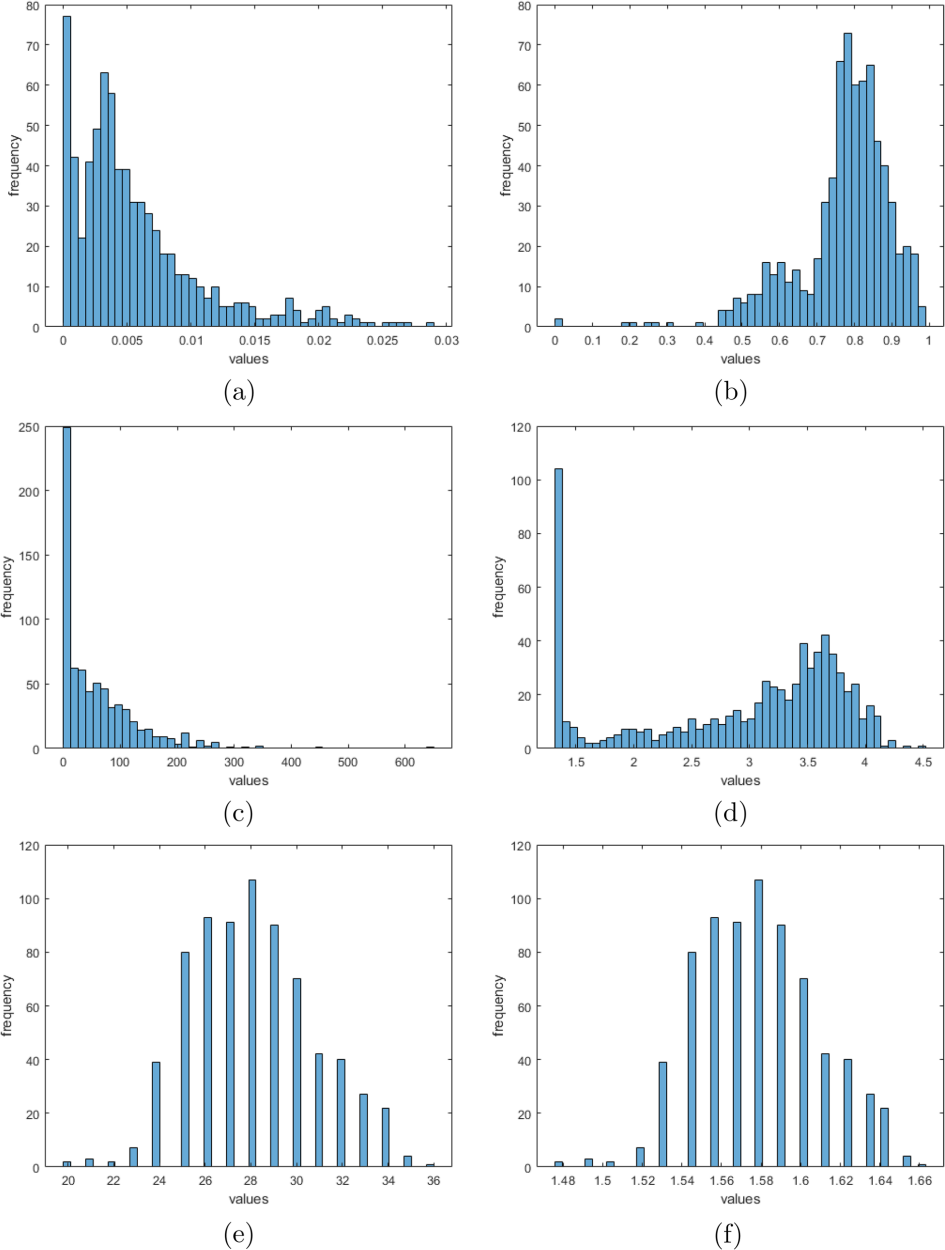
Distribution of the incidence, rainfall, and temperature; **a**, **c** and **e** for original data and **b**, **d** and **f** for transformed data respectively

We used the parameter values for [shift, div] as [23, 2e-2] and [10, 1] for rainfall and temperature data transformation respectively. The visualisation of the data transformation is presented in Fig. 4.

The primary statistical analysis, involving panel regression modeling (see Panel regression section), was conducted using STATA software. This analysis focused on exploring the relationship between malaria incidence levels and weather variables while accounting for panel data structure and individual-specific effects. The preliminary analyses, such as cross-correlation and autocorrelation assessments (see Cross-correlations and autocorrelations section), were performed using MATLAB software. These initial analyses helped identify correlations and patterns in the data, providing insights into the relationship between variables before proceeding with more advanced modeling techniques in STATA. Additionally, the PSO clustering technique used for clustering the incidence data was performed in MATLAB.

## Results

### Incidence-weather cross-correlations and incidence-specific autocorrelation

**Fig. 5:**
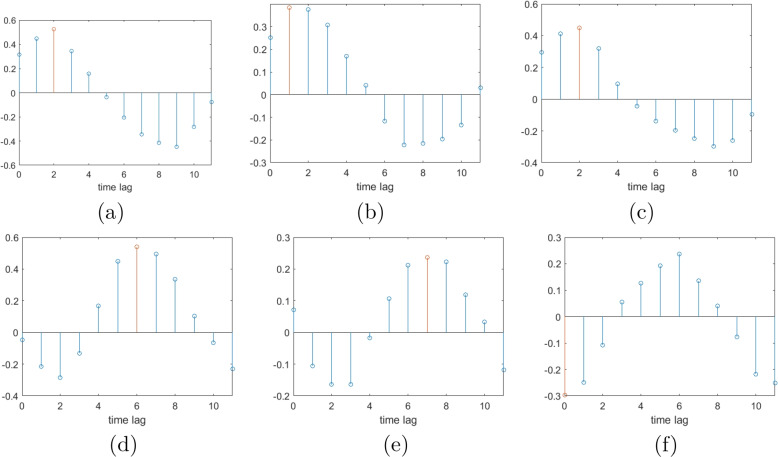
Example cross-correlation results for 3 states: **a** Gombe incidence-rain cross-correlation **b** Delta incidence-rain cross-correlation **c** Kebbi incidence-rain cross-correlation **d** Gombe incidence-temperature cross-correlation **e** Delta incidence-temperature cross-correlation **f** Kebbi incidence-temperature cross-correlation

We observed that the correlations between malaria incidence and rainfall predominantly exhibit initial positive coefficients, which gradually transition to negative coefficients as the lag duration increases (see Fig. 5a-c). Conversely, the correlations between malaria incidence and temperature demonstrate an opposite trend (see Fig. 5d-f). The time lags associated with the highest correlation coefficients between malaria cases and rainfall typically range from 1 to 3 months, with a majority occurring at a 2-month lag (see Table 1). This conforms with previous studies that have found approximately a two-month lag between the peaks of rainfall and malaria incidence [46–48]. However, for the correlations between malaria incidence and temperature, the maximal correlation coefficients exhibit a wide variability, ranging from 0 to 7 months, and encompass both negative and positive values (see Table 1). This fluctuating pattern of cross-correlations may be attributed to the monthly data collection frequency, which represents a relatively large time scale for measurement.

In our study, lag values obtained from the cross-correlation analysis for rainfall are used to construct appropriate variables in the regression models, while those for temperature are excluded due to their unstable nature.

**Table 2:** Spearman autocorrelation matrix

Lag	0	1	2	3	4	5	6
0	1						
1	0.58545	1					
2	0.63888	0.58545	1				
3	0.45374	0.63888	0.58545	1			
4	0.34347	0.45374	0.63888	0.58545	1		
5	0.37605	0.34347	0.45374	0.63888	0.58545	1	
6	0.19333	0.37605	0.34347	0.45374	0.63888	0.58545	1

Table 2 shows the case-specific auto-correlations with the Spearman-rank correlation coefficient. This information can be useful in understanding the temporal patterns and potential predictive power of past malaria incidence on current cases. The results in Table 2 suggest there is a positive correlation between malaria incidence in the current month and the incidence in the previous months, with varying strengths depending on the lag. The correlation coefficient of 0.63888 at lag 2 suggests a relatively strong positive correlation between malaria incidence in the current month and the incidence two months ago. The coefficient at lag 6 is 0.19333, indicating a relatively weak positive correlation between the current month and the incidence six months ago. This is followed by the coefficient of 0.34347 at lag 4 which suggests a weaker positive correlation between the current month and the incidence four months ago.

### Clustering

The model featuring cluster-specific effects yields improved outcomes (in terms of increased $R^2$ value and minimized RMSE) when utilizing the arbitrarily chosen cluster barriers, as opposed to when the clustering strategy is not employed [49]. Nevertheless, investigation is undertaken to ascertain whether clustering by dividing into tertiles represents the optimal approach, or if a different set of cluster barriers can outperform it in terms of minimizing the mean square error. Therefore, the optimal lower barrier $b_l$ and upper barrier $b_u$ are sought using the method of particle swarm optimization (PSO) [39, 44]. The PSO technique is a metaheuristic algorithm that does not rely on gradient information and utilizes a stochastic approach to converge toward optimal solutions. Specifically, the PSO variant employed in this study involves updating players’ positions (barriers) and velocities iteratively, with parameters such as self-confidence (1.0), global-best position attraction (1.0), inertia (1.0), and constriction factor (0.3) carefully tuned for optimal performance. The algorithm utilized 100 players to form a relatively large swarm, balancing between exploration and exploitation. Further details on the implementation can be found in [39].

**Fig. 6:**
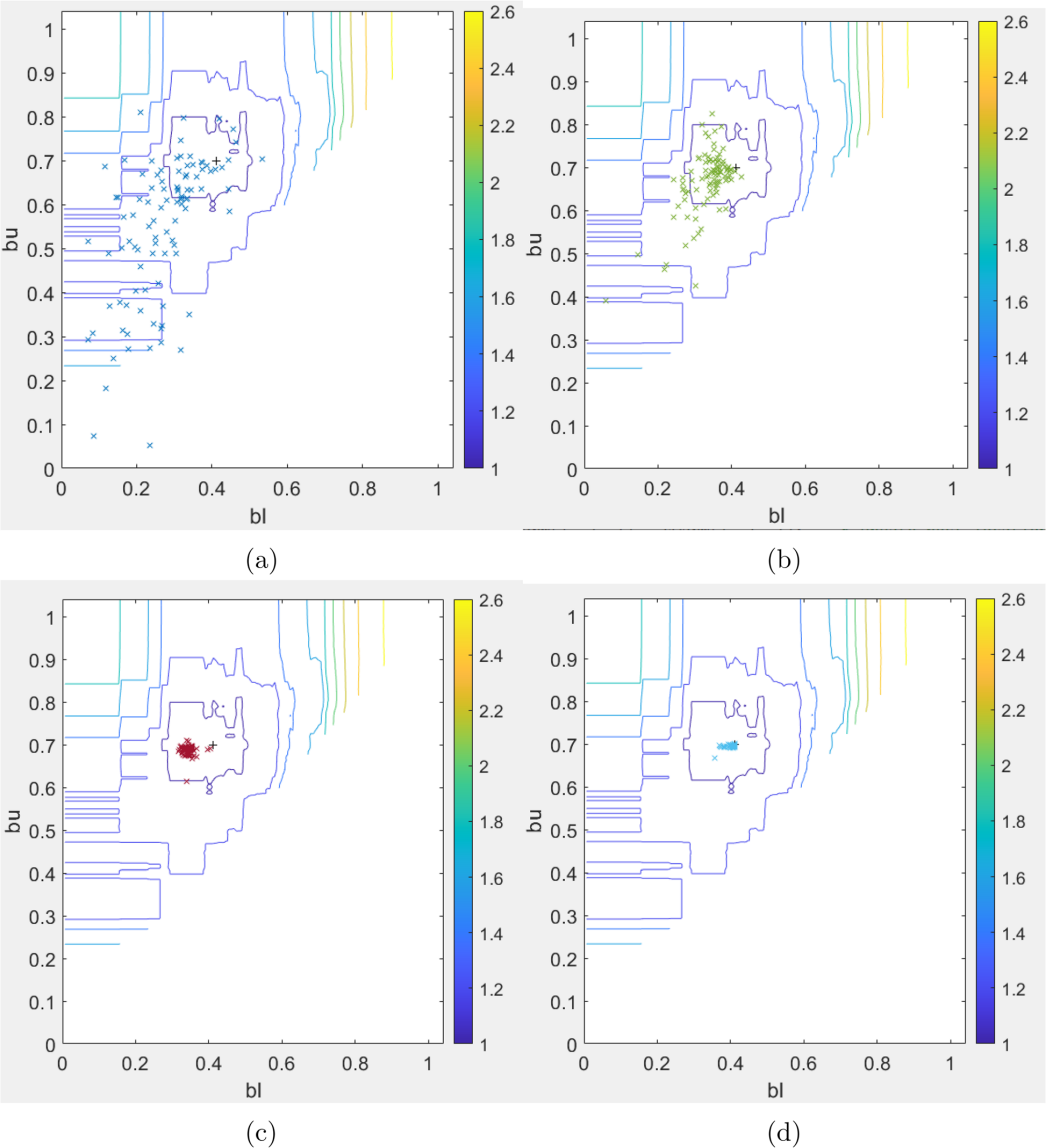
Computation of optimal barriers ($b_l, b_u) \approx (0.4124, 0.6990)$ for the clustering. Black ($+$) denotes the optimal barriers determined by the PSO computation. The figures show the evolution of the positions of 100 players ($\times$) converging to an optimal solution: **a** 5th iteration **b** 10th iteration **c** 20th iteration **d** 40th iterations

The optimal barrier values are ($b_l=0.4124,b_u=0.6990$). The surface plot of the MSE with respect to the barriers $b_l$ and $b_u$ is presented in Fig. 6.

**Fig. 7:**
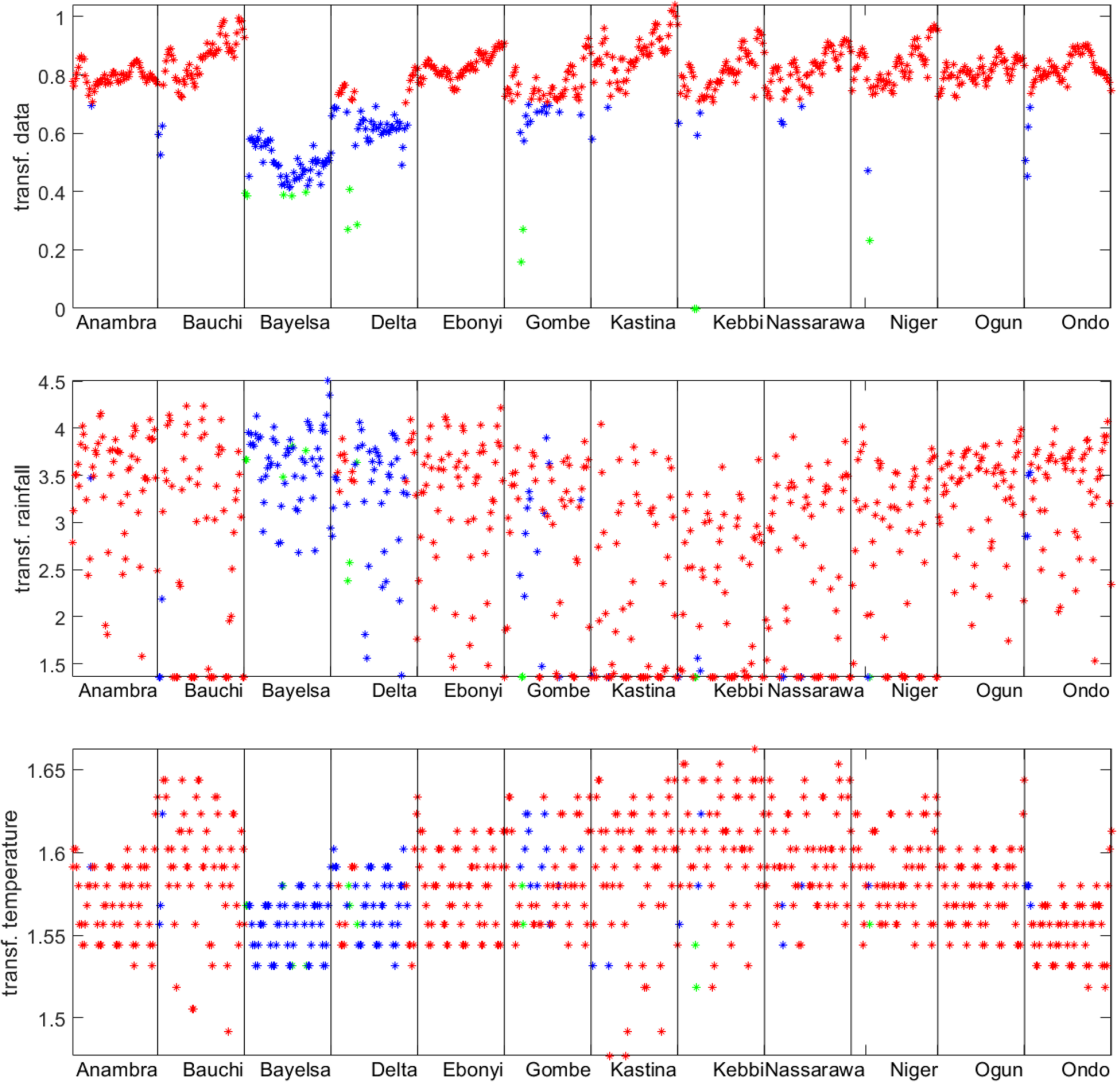
Malaria data and the meteorological data after clustering with optimal barriers

The malaria data and the meteorological data after clustering with optimal barriers are given in Fig. 7 .

### Panel regression models

We investigated variable selection for model specification, considering criteria such as fit, complexity, insignificance, negative marginal effects, and multicollinearity stemming from certain variables. For fit and complexity evaluation, we aimed to minimize the Bayesian Information Criterion (BIC) [43, 50]. The BIC incorporates a likelihood function *L* and penalizes the number of parameters (*k*) more heavily compared to the Akaike Information Criterion (AIC) [51], particularly for large observation sizes, by including a term proportional to $\log (N)$, where *N* represents the sample size. Our objective was to reduce BIC by eliminating certain variables and addressing issues of insignificance and multicollinearity as well.

Significance testing was performed using the standard *t*-test, while multicollinearity assessment involved computing the Inverse Variance Inflation Factor (1/*VIF*) for all explanatory variables except the constant term. A 1/*VIF* value below the threshold of 0.1 indicates multicollinearity associated with the tested variable [52]. Additionally, we monitored the *p*-value of the *F*-statistic, which indicates whether the overall set of variables is jointly significant; a *p*-value smaller than the significance level $\alpha =0.05$ suggests significance. This approach not only evaluates the model’s adequacy beyond a constant term model but also helps diagnose multicollinearity.
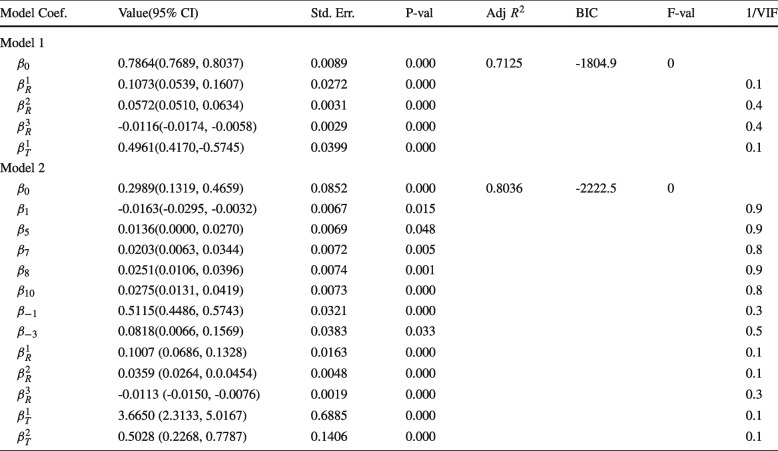


In this study two different models were examined: the first model (Model 1) featured incidence clustering with optimal barriers, while the second model (Model 2) incorporated lag incidence cases and individual-specific effects. The emergence of individual-specific effects automatically categorizes the model as either a fixed-effect or a random-effect model. We opted for a random-effect model to account for variability not explicitly addressed by the model variables, a choice also supported by a Durbin-Wu-Hausman test.

The model including lag incidence cases and individual-specific effects exhibited the highest Adjusted $R^2$ Value (0.8036) and lowest BIC value (-2222.5), outperforming the model solely based on incidence clustering, which had $R^2$ and BIC values of 0.7125 and -1804.9 respectively. Model 2 highlights the significance of individual-specific effects on malaria incidence in five cities (Anambra, Ebonyi, Kastina, Nassarawa, and Ogun), indicating meaningful variability in malaria incidence outcomes specific to these cities beyond what can be explained by observed explanatory variables (rainfall and temperature).

Then, we checked if certain marginal effects would be consistent with our auto-correlation study. From Table 2, it is seen how cases in the past 6 months positively predict present cases with the least auto-correlations found from cases from the last 4 to 6 months. The case-specific auto-correlation supports the model specification where lag-1 to lag-3 incidence are significant predictors for present incidence, whereas lag-2 incidence was omitted due to negative marginal effects that may have resulted from certain model specifications.

In both models, all marginal effects corresponding to the rainfall and temperature matrices are positive, except for the effect of rain in the upper cluster, which exhibited a negative marginal effect. This suggests that higher rainfall leads to lower incidence in the upper cluster. In Model 2, both rainfall and temperature have the highest marginal effect in the lower cluster and the least effect in the upper cluster. This pattern is also observed for the marginal effects with respect to rainfall in Model 1, whereas temperature significantly predicts only incidence in the lower cluster. For both models, temperature could not significantly predict cases in the upper cluster.

**Fig. 8:**
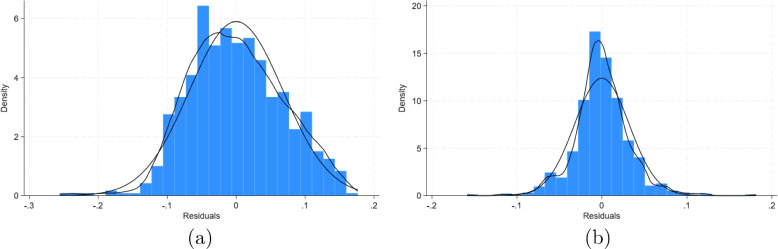
Residual plots for **a** Model 1 **b** Model 2

**Fig. 9:**
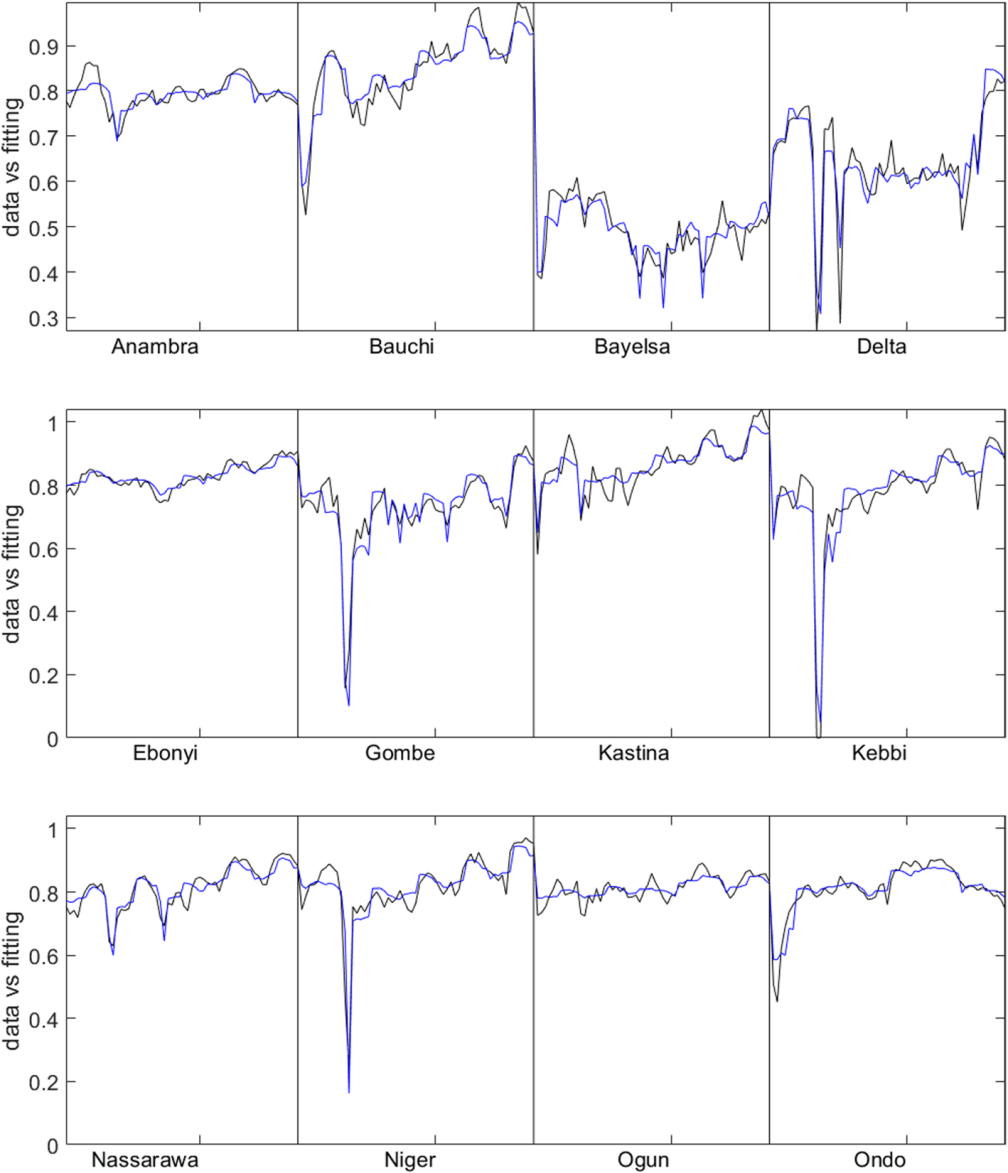
Fitting result (blue lines) for Model 2

We also checked that residuals of the models follow a normal distribution which is crucial for ensuring the validity, adequacy, and reliability of the associated inferences. It can be seen from Fig. 8 that the residuals of both models follow a normal distribution with Model 2, conforming slightly better than the first model. The plot of the model fits with the data for Model 2 is given in Fig. 9.

## Discussion

We employed a clustering approach to partition datasets into multiple subsets, not only enriching the explanatory variables but also accurately incorporating the role of weather in predicting specific ranges of incidence data. The clustering-integrated regression models were complemented by optimal barriers. Given that varying the clustering barriers returns different modeling results, we aimed to identify optimal barriers that minimize the mean squared error.

In this study, insights from cross-correlations and autocorrelations between weather factors (rainfall and temperature) and malaria incidence were utilized to incorporate suitable variables in the regression models.

Two models were deliberated: clustering-integrated models with and without lag incidence and individual-specific effects. The selection of the model, along with its implications (marginal effects), hinges on the decision-maker’s priorities. When $R^2$ and BIC are of paramount importance, we advocated for the clustering-integrated model with lag incidence cases and individual-specific effects. Notably, the significance of certain individual-specific effects suggests substantial variability in malaria incidence outcomes specific to these five cities, beyond the explanatory capacity of observed variables (rainfall and temperature). Indeed factors, such as mosquito breeding site availability and human behaviors (e.g., healthcare-seeking practices, bed net usage), can influence these effects [53, 54].

In the model, all marginal effects related to rainfall and temperature matrices exhibit positivity, except for rainfall’s effect in the upper cluster, which displays a negative marginal effect. This suggests that higher rainfall correlates with lower incidence in the upper cluster, reflecting the intricate and context-dependent relationship. Indeed, high rainfall is known to eliminate mosquito breeding sites [21, 22]. The clustering-integrated model solely comprising weather components is preferable when weather takes precedence over lag incidence cases or in scenarios where data on individual-specific effects are lacking.

In Model 2, both rainfall and temperature exert the highest marginal effect in the lower cluster and the least effect in the upper cluster. This pattern is consistent with Model 1’s marginal effects concerning rainfall, whereas temperature significantly predicts incidence only in the lower cluster. Interestingly, temperature fails to significantly predict cases in the upper cluster, suggesting physical implications where rainfall can predict incidence cases consistently throughout the year, while temperature can only predict low-to-medium incidence scenarios. Thus, this research suggests that while temperature and rainfall may influence disease incidence under certain conditions, their predictive power varies depending on the severity of the outbreak or other contextual factors.

The increasing demand for confounding factors to explain various incidence levels is mitigated by incidence clustering. This approach supports the notion of considering specific hypothetical factors for predicting malaria incidence and conventional regression modeling with limited explanatory variables [38, 39, 55]. The localization of accurately predicted incidence via weather components bears significant implications for public health authorities, not only informing the extent of prediction through marginal effects but also facilitating proactive measures amidst impending weather changes.

Ultimately, the present study highlights the importance of compiling data on additional confounding factors (e.g., other weather components, bednet availability and usage, presence of stagnant water bodies,etc), which not only introduce more explanatory variables but also enhance the reliability of the analysis.

## Data Availability

All the data sources have been mentioned. The datasets used and/or analyzed during the current study are available from the corresponding author upon reasonable request.
